# The long walk to a short half-life: the discovery of augmented renal clearance and its impact on antibiotic dosing

**DOI:** 10.1093/jac/dkaf378

**Published:** 2026-06-12

**Authors:** Jeffrey Lipman, Russell E Lewis

**Affiliations:** Jamieson Trauma Institute, Royal Brisbane and Women’s Hospital, and Queensland University of Technology, Brisbane, Australia; Anaesthesiology and Critical Care, The University of Queensland, Brisbane, Australia; UR-UM 103, University of Montpellier, Division of Anesthesia, Critical Care, Emergency and Pain Medicine, Nîmes University Hospital, Nîmes, France; Department of Molecular Medicine, University of Padua, Padua, Italy

## Abstract

**Background and objectives:**

Renal function monitoring traditionally focuses on detecting impairment to prevent antibiotic toxicity. However, augmented renal clearance (ARC) represents the opposite challenge—enhanced elimination causing subtherapeutic drug concentrations. The aim of this review is to describe ARC’s discovery and its impact on antibiotic therapy over two decades.

**Methods:**

Narrative commentary examining ARC’s discovery, clinical significance, diagnostic challenges and management strategies for antibiotic dosing in critically ill patients, with future research priorities.

**Results:**

ARC was first noted in the late 1990s at Baragwanath Hospital, South Africa, where unexpectedly high creatinine clearance rates (>200 mL/min) were measured in ICU patients. Subsequent pharmacokinetic studies confirmed elevated antibiotic clearance with reduced systemic exposures. ARC, defined as creatinine clearance of >130 mL/min/1.73 m^2^, occurs in 65%–80% of critically ill patients with normal serum creatinine, particularly younger patients with sepsis, trauma or burns. The phenomenon results from increased cardiac output and renal blood flow during systemic inflammatory responses, can persist for weeks after ICU admission, and affects all renally eliminated drugs. ARC is often undiagnosed unless some form of creatinine clearance is directly measured. Importantly, ARC is a major risk factor for antibiotic failure and resistance selection.

**Conclusions:**

ARC represents a significant but underrecognized challenge affecting antibiotic dosing in critically ill patients. Therapeutic drug monitoring remains the most reliable method to ensure adequate antibiotic exposure. Future research priorities include validated predictive models, simpler diagnostic methods and evidence-based dosing guidelines for high-risk populations.

## Introduction

Antibiotic dosing has traditionally focused on renal impairment and dose reduction to prevent drug accumulation and toxicity. Augmented renal clearance (ARC)—defined as creatinine clearance exceeding 130 mL/min/1.73 m^2^ despite normal serum creatinine levels—presents the opposite challenge: enhanced drug elimination leading to subtherapeutic concentrations and potential treatment failure. ARC occurs in a large proportion of critically ill patients with normal serum creatinine levels, particularly younger individuals with sepsis, trauma or burns. Despite affecting two-thirds of this population, ARC often remains underrecognized, creating substantial challenges for optimal antibiotic therapy in intensive care settings where standard dosing may result in poorer outcomes and contribute to antimicrobial resistance. This commentary examines the discovery of ARC and two decades of research on its impact on antibiotic dosing.

## The discovery of ARC

In the 1980s, the multidisciplinary ICU at Baragwanath Hospital in Soweto, South Africa (now Chris Hani Baragwanath Academic Hospital) faced a logistical challenge that would ultimately lead to an important clinical discovery. The hospital, a sprawling single-storey facility encompassing 72 wards across 73 acres, maintained its pathology services in a centralized laboratory located approximately 1–1.5 km from the ICU. Despite having automated computer-based results reporting, the institution lacked an automated specimen collection system. Daily routine blood samples were collected from patients at predetermined intervals and deposited in central ward areas for pick-up by pathology clerks. However, these personnel transported specimens to the laboratory by foot, with the lengthy walk often interrupted by smoking breaks and social interactions along the way, resulting in delays and occasional sample loss that necessitated repeat collections.

Recognizing the inefficiencies of this system, the ICU leadership decided to place a dedicated electrolyte analyser (Beckman Coulter clinical chemistry analyser) within the ICU building. A technician was also hired for sample analysis and was cross-trained with the existing ICU equipment technician to provide consistency in both analytical and technical support capabilities. The implementation of in-house analytical capabilities enabled daily measurement of electrolytes and, following resolution of quality control protocols, expanded to include creatinine analysis in both plasma and urine samples. Routine urine creatinine measurement soon became a standard of care in the ICU for all patients with indwelling urinary catheters.

The ICU team established a protocol for daily creatinine clearance calculations using the standard UV/P equation (urine creatinine concentration × timed urine volume ÷ plasma creatinine concentration). An 8 h urine collection period was used from midnight to 8:00 AM, with corresponding blood sampling performed between 4:00 AM and 6:00 AM. This systematic approach of measuring timed urine collections soon revealed creatinine clearance values of 200 mL/min or higher in some patients, particularly among younger patients. These unbelievably high clearance rates raised concerns regarding assay accuracy, measurement technique or analytical procedures, as established literature-defined ‘normal creatinine clearance’ ranged between 60 and 130 mL/min.^1^ Results of 200 mL/min seemed impossible.

**Figure 1.**
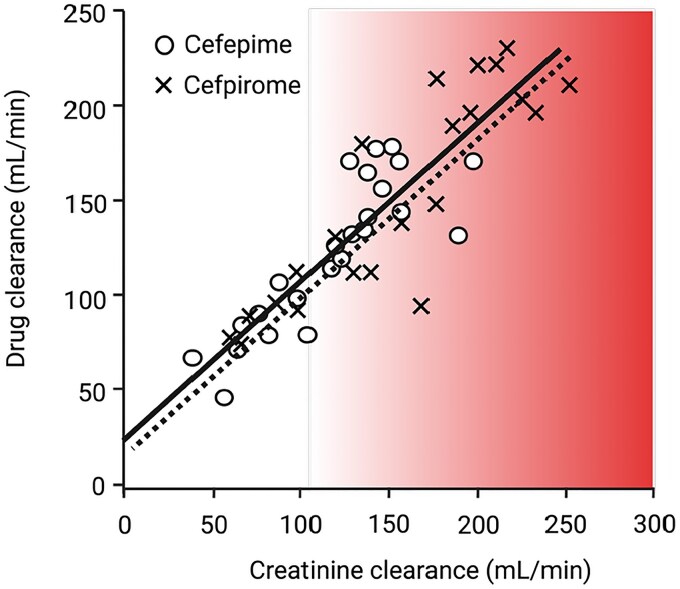
Relationship of cefepime and cefpirome clearance versus measured 24 h creatinine clearance in ICU patients. The linear relationship between measured 24 h creatinine clearance (*x*- axis) and calculated drug clearance (*y*-axis) is represented for cefepime (solid line) and cefpirome (dotted line). Red shading indicates a region of patients classified with ARC. Figure is adapted from Lipman *et al*.^4^

By the late 1990s, however, investigators became more convinced that extremely high creatinine clearance rates were a real clinical problem in the ICU. Lipman (by now in Australia) and colleagues incorporated timed creatinine clearance measurements as part of their pharmacokinetic studies of two renally eliminated fourth-generation cephalosporins.^2–4^ Their data confirmed that elevated clearance rates—previously assumed to represent measurement artefacts—were associated with significantly higher clearance rates for cephalosporins and reduced serum drug exposures (Figure 1). This observation had profound implications for antibiotic dosing, suggesting a fundamental reassessment of pharmacokinetic principles of dosing in ICU patients.^5–7^

Subsequent studies from Lipman’s group further identified common pathophysiological and iatrogenic contributors to the elevated creatinine clearance phenomena.^8,9^ Changes in organ function can be dramatic in ICU patients, as a consequence of underlying pathologies, as well as in response to clinical interventions. With the common fluid administration in sepsis (plus often inotropic use), as well as the intrinsic increases in cardiac output and major organ blood flow in the pathophysiology of the inflammatory response of some of these patients, the clearance of many commonly prescribed agents, particularly those that are cleared through the kidneys, resulted in subtherapeutic drug levels and potential treatment failure if serum concentrations (or tissue concentrations) were not maintained at certain levels.

**Figure 2.**
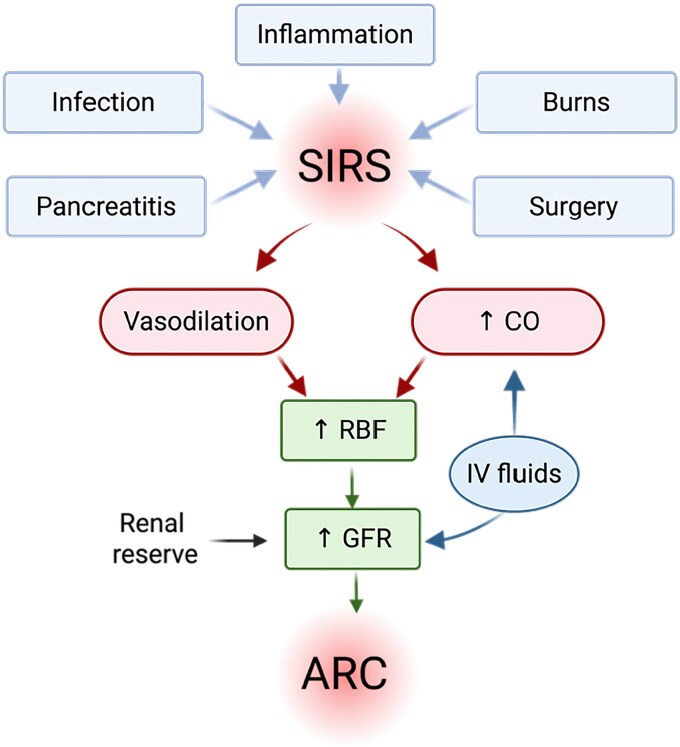
Pathophysiology and clinical factors that contributed to ARC. SIRS, systemic inflammatory response syndrome; ↑CO, increase in cardiac output; ↑RBF, increase in renal blood flow; ↑GFR, increase in GFR. Adapted from Udy AA *et al*.^6^ Created in BioRender by R.L. (2025; https://BioRender.com/k0ysqiv).

Lipman and colleagues began to systematically study this elevated creatinine clearance phenomenon in critically ill patients even though higher-than-normal clearance via glomerular filtration had been reported previously.^10^ Lipman’s group coined the term ‘augmented renal clearance (ARC)’ to describe a phenomenon that arises from a ‘standard inflammatory response’ of conditions that lead to high cardiac output and increased renal blood flow—producing elevated glomerular filtration rates (GFR) associated with high creatinine clearances (Figure 2).^6,8,9^

Initially, 8–24 h urine collections were used to measure timed creatinine clearances, but shorter collection periods may be effective for detecting ARC, with intervals as brief as 2 h demonstrating comparable utility.^11^ However, shorter collection periods amplify the impact of timing errors through mathematical extrapolation. For example, when a 2 h collection is extrapolated to 24 h (multiplied by 12), even minor inaccuracies in collection timing translate into substantial errors in the calculated clearance rate. Consequently, precise timing becomes critical when using abbreviated collection protocols, requiring meticulous attention to start and stop times to ensure accurate measurements.

## How prevalent is ARC?

ARC is a phenomenon resulting in unexpectedly high renal excretion of solutes, including many commonly used medications and, more specifically to this manuscript, antibiotics, as well as some antifungals and antivirals.^2–7,12–22^ ARC therefore can lead to subtherapeutic drug concentrations, potentially resulting in treatment failure, increased morbidity and mortality, and the development of antimicrobial resistance. ARC once occurring in the ICU often lasts for a week or more, with one study mentioning that ARC can last for more than a month.^23^ While many studies have defined the prevalence of ARC based on timed creatinine clearance, other studies have defined the population of patients with ARC based on low systemic exposures of antibiotics in patients with ‘normal’ serum creatinine. Lipman and colleagues established a threshold creatinine clearance of 130 mL/min/1.73 m^2^ to define ARC,^6^ a cut-off that has been widely adopted in subsequent research (Table 1). However, variable definitions of ARC persist throughout the literature, likely contributing to the substantial variability in reported incidence rates across different ICUs.

**Table 1.** Key studies describing epidemiology and risk factors of ARC in adult and paediatric populations

Authors, year	Study design	Reported incidence of ARC	Identified risk factors and clinical consequences
Fuster-Lluch *et al.*, 2008^10^	Prospective, observational study in adult ICU population	Incidence: Glomerular ‘hyperfiltration’ of 17.9%–30% during first week of ICU care	Risk factors: 75% of patients with albuminuria Consequences: Further studies in drug elimination are required to guide dosing
Udy *et al.*, 2013^24^	Prospective, observational study in adult ICU population	Incidence: 57.7% overall, 85.7% in trauma patients 39.5% in septic patients	Risk factors: Younger age, male gender, lower APACHE II and SOFA scores, higher cardiac indices Consequences: Potential for sub-therapeutic drug exposure, increased clinical failure or drug resistance with β-lactam antibacterial therapy Need for TDM and higher antibiotic dosing
Carlier *et al.*, 2013^25^	Prospective, observational pharmacokinetic study in adult ICU population	Incidence: 48% of patients had ARC Range 30%–85% during ICU care	Consequences: Among 48% of patients with ARC, 76% of these did not achieve the pharmacokinetic target of 100% *fT*_>MIC_
Claus *et al.*, 2013^26^	Prospective observational study in adult ICU	Incidence: 51.6% 12% persistent ARC	Risk factors: Younger age and male gender Consequences: Worse outcome with antibiotic treatment
Udy *et al.*, 2014^27^	Multicentre, prospective, observational study in adult ICU	Incidence: 65.1% manifested ARC on at least one occasion during the first 7 study days; Majority (74%) of whom did so on ≥50% of their creatinine clearance measure	Risk factors: Younger age (trauma), systemic inflammation Consequences: Renally eliminated drugs: low molecular weight heparins, aminoglycosides, glycopeptides and β-lactams) at risk for subtherapeutic concentrations and potentially adverse clinical outcomes
Udy *et al.*, 2017^28^	Sub-study of the BLING-II trial, which aimed to explore the association between ARC and patient outcomes in a large randomized clinical trial	Incidence: 17.7% in patients with traumatic brain injury Present throughout the ICU stay, as data were collected until ICU discharge, death or Day 15.	Risk factors: Cardiovascular changes and elevated plasma atrial natriuretic peptide (ANP) concentrations Consequences: Lower exposures to renally eliminated drugs, including levetiracetam
Baptista *et al.*, 2020^29^	A retrospective cohort study in adult ICU patients	Incidence: 24.9%	Risk factors: Trauma, young age and male sex
Dhaese *et al.*, 2021^30^	Prospective observational study in adult ICU patients with COVID-19 infection	Incidence: 72% (95% CI 64%–79%). The proportion of ARC days was 15.6 (14.1–17.3) per 100 ICU days. The median number of ARC days per patient was 2 Median (IQR) first day of ARC was Day 2 (3–5) of ICU stay ARC days per 100 ICU days is lower when compared to a general ICU population (15.6 versus 36.6 per 100 ICU days)	Risk factors: Younger COVID-19 patients are at increased risk of ARC Consequences: A low threshold for measuring creatinine clearance and, if available, TDM of renally eliminated drugs in critically ill COVID-19 patients is advisable
Andre *et al.*, 2021^31^	Retrospective pharmacokinetic evaluation of meropenem and piperacillin exposure in paediatric haematology/oncology patients	Incidence: Nearly two-thirds of samples from children with ARC Episodes of ARC reported in 30%–40% of children with malignancies	Risk factors: Hypermetabolic state due to malignant cell multiplication and tumour cell lysis after chemotherapy Clinical impact: Insufficient antimicrobial exposure in 85% of cases despite high dosages; need for TDM and potentially higher antibiotic dosing; modifying treatment modalities such as prolonged infusion times or continuous infusion for piperacillin
Wang *et al.*, 2023^32^	Retrospective pharmacokinetic review evaluation of vancomycin TDM results in paediatric haematology/oncology patients	Incidence: 79.7%	Risk factors: Age-associated levels of serum creatinine, younger age (especially less than 8 years), aplastic anaemia more than in those with other haematological diseases due to a higher BMI and a lower GFR Consequences: Suboptimal vancomycin trough concentrations, need for TDM, potential need for higher antibiotic dosing, especially in children younger than 8 years
Mikami *et al.*, 2023^23^	Retrospective study in mixed ICU population to develop risk prediction model	Incidence: 32% in the training set 40% in the validation set	Risk factors: High creatinine clearance, muscle mass loss affecting serum creatinine levels Consequences: JPNARC score was developed to predict ARC onset and showed some predictive utility in identifying patients at risk for ARC
Cook *et al*., 2024^64^		Incidence: 77.3% in TBI Duration: persisted throughout the seven-day study period	Hyperdynamic state following acute trauma, which includes alterations in stress hormone concentrations, vascular tone, fluid status, cardiac output and altered blood flow to major organs Significantly lower levetiracetam concentrations, which may lead to reduced efficacy in preventing seizures and increased treatment failure
Ye *et al.*, 2025^33^	Retrospective observational study in neurocritical ICU	Incidence: 56.2%	Risk factors: Highest prevalence in patients with spinal lesions (3/4), followed by TBI (64.5%), cerebrovascular diseases (53.5%), cranial tumours (51.0%) and intracranial infection (50%) Consequences: Activation of neural–renal axis, increased excretion rate of renally eliminated drugs

TBI, traumatic brain injury.

ARC has also been documented in paediatric patients,^31,34–39^ with the same drug dosing implications and clinical consequences of underdosing.^31,37,38^ Serum creatinine use for estimated GFR (eGFR) is commonly performed in paediatric patients, noting though that serum creatinine varies with age and sex, particularly during early childhood (aged <2 years). Accurately measuring GFR in children is problematic, often with the need of external markers such as iohexol or cystatin C, combined with blood sampling to determine concentration–time curves.^40,41^

These problems are exacerbated by the lack of the common practice of adult ICU patients having an *in situ* urinary catheter. Nevertheless, due to lack of kidney problems in paediatrics, the overall prevalence of ARC in this population is greater than in adults.

The actual prevalence, however, in paediatric patients is less well defined versus the adult ICU population. ARC has also been increasingly recognized in specific patient groups outside the ICU,^42^ including those with severe infections like COVID-19^30^ and patients with febrile neutropenia.^43^

## Diagnosis of ARC

ARC is undiagnosed unless some form of creatinine clearance is directly measured (either via a timed measurement of urine creatinine or less accurately from equations using serum creatinine; see comments below). Although the reported incidence varies in the literature (see Table 1), Lipman and colleagues reported ARC in up to 65% of adult ICU patients with a ‘normal’ serum creatinine.^27^ Others have reported prevalence rates as high as 80% in adult ICU patients with ‘normal’ serum creatinine.^44^ The overall percentage is about 30%^22,23^ However, this will vary across ICUs according to admission criteria of each specific unit; surgery and trauma admissions will have a higher prevalence than those of medical ICUs with young surgical patients, particularly neurosurgical trauma having a large inflammatory response to trauma with kidneys being ‘normal’ (see Table 1).

Identifying ARC requires a high index of suspicion and appropriate renal function assessment. The gold standard for diagnosing and quantifying ARC is through timed urine collections, typically over an 8 or 24 h period. Estimation from equations, e.g. Cockcroft–Gault or CKD-Epi, while convenient, often underestimate true renal function in patients with ARC and should be used with caution^45^ as these equations were created for patients with renal dysfunction. They may serve as initial screening tools, but confirmation (at least once) with a measured creatinine clearance is often necessary.^42,45^

There have been attempts to use scoring systems to calculate the impact of ARC,^24,29,46–48^ but these are merely ARC prediction scores and should be used as such. They are neither designed for, nor are substitutes for, actual creatinine clearance measurements to quantify the ARC danger to dosing.^42,45^

Quantifying morbidity and mortality issues in patients with ARC is complicated, as on the one hand, ARC can lead to treatment failure, but on the other hand, ARC occurs in a young patient group with fewer underlying comorbidities.^26,28^

## Management of ARC

The cornerstone of managing ARC is the adjustment of drug regimens to ensure therapeutic drug exposure. The primary concern with ARC is its impact on the pharmacokinetics of renally eliminated drugs. As the quantification of ARC is not straightforward, unrecognised ARC is a danger to dosing. When renal clearance is significantly elevated, drugs that are primarily excreted by the kidneys are removed from the body at an accelerated rate. This can lead to subtherapeutic drug concentrations.^2,5–7,12–14,16,19,34^ Standard drug dosages may not achieve or maintain the necessary therapeutic levels in patients with ARC. This is particularly critical for time-dependent antibiotics, i.e. β-lactams, which require sustained concentrations above the MIC for efficacy.^49^ Potentially this can lead to treatment failure, leading to prolonged illness, complications and increased length of hospital stay. The most effective strategy to mitigate this risk of therapeutic failure is the implementation of therapeutic drug monitoring (TDM) for β-lactam antibiotics.^50^ Although TDM remains underutilized in clinical practice, it currently represents the most reliable method to ensure adequate antibiotic exposure in patients with ARC. Other potential complications of ARC are antimicrobial resistance due to suboptimal antibiotic concentrations, which promote the selection and proliferation of resistant bacterial strains, a major public health concern.

The clinical significance of ARC may vary by infection site, with potentially greater impact on treatment outcomes in infections where β-lactam penetration may be reduced (e.g. ventilator-associated pneumonia) versus infection sites with inherently higher concentration of drug, such as urinary tract infections.

ARC also impacts the pharmacokinetics of renally eliminated antifungals and antivirals.^49,51^ All renally excreted drugs have the danger of being underdosed when ARC is present, and after ICU admission, ARC may persist for several weeks.^27^ Ideally, a form of drug level monitoring, e.g. TDM, should be used whenever possible for all drugs renally eliminated in patients with ARC.

Optimized antibiotic dosing strategies have also been proposed to address ARC. Continuous infusion of β-lactam antibiotics has gained renewed attention as a potential solution, given the theoretical advantage of maintaining steady-state drug concentrations.^52,53^ Whilst continuous or prolonged infusions may improve some aspects of antibiotic underexposure exacerbated by ARC and renal clearance of antibiotics,^25,28,54–57^ neither strategy completely eliminates the risk of subtherapeutic drug exposure.^58^ Currently, routine TDM is recommended for vancomycin, teicoplanin, aminoglycosides, voriconazole, β-lactams and linezolid in critically ill patients.^59,60^ Newer approaches, such as model-informed precision dosing (MIPD), offer individualized dosing regimens designed to achieve target pharmacokinetic/pharmacodynamic indices based on multiple patient covariates available before the first dose. Through Bayesian estimation after TDM results are available, dosing regimens can be further optimized using patient-specific pharmacokinetic parameters to maximize the probability of achieving therapeutic targets.^61^ However, careful model selection is essential, considering factors such as patient population (age, clinical setting, comorbidities), body composition, dosing ranges and analytical methods used in model development. Critically, many existing models were not developed specifically in populations with ARC or do not capture dynamic changes in renal function,^62^ therefore validation of predictive performance in ARC patients will be essential before clinical implementation.

A detailed review of ARC by Sistanizad and colleagues^12^ provides recommendations for antibiotic dosing regimens in patients with ARC.

## Challenges and future directions

Future research on ARC should focus on three critical areas. First, development of more accurate predictive models to identify patients at risk for ARC. Second, establishment of simpler diagnostic methods that do not require timed creatinine clearance measurements, which remain impractical in many clinical settings. Third, creation of evidence-based dosing guidelines for the numerous renally eliminated medications used in this patient population.

The development of more accurate models for predicting which patients will develop ARC would be a major advance. Currently, the diagnosis and identification of ARC is with the use of a timed urine measurement of volume and creatinine in it, with concurrent measurement of plasma creatinine (UV/P). Iohexol has been used to estimate GFRs but is expensive^63^ and not widely available. There is some interest in cystatin C to estimate GFR in renal dysfunction although use for ARC, whilst limited, is increasing.^33,64^ Standard creatinine-based equations are commonly used but these are unreliable. It would be helpful if some type of easily identified, cheap biomarker could be used to identify patients with ARC, although it seems that creatinine clearance is the main method for the near future. Currently, the proposed ARC scoring systems have not been widely validated^24,29,46–48^ and are considered to have limited clinical utility to guide decisions in clinical care.

Enhanced quantitative assessment of ARC severity could enable more precise antibiotic dosing without requiring frequent creatinine clearance measurements. An accessible and cost-effective biomarker that accurately predicts the extent of ARC would significantly improve dosing precision in this population. The absence of such tools represents a major clinical challenge, as appropriate dosing in patients with ARC remains extremely difficult without TDM or other direct measurements of drug exposure.^13,15–20,22,39,49,50,65^

Ultimately the goal should be to develop reliable, real-time tools for the diagnosis of ARC, and quantify the severity of the phenomenon to assist in dosing strategies.^62^ This would entail some sort of renal clearance biomarker^33,39,40,63,64^ to accurately predict the resulting dosing requirements, eliminating the current danger to dosing.

Current prediction models demonstrate limited accuracy for patients with ARC because the populations used to develop these models contained insufficient numbers of patients with ARC. Most predictive algorithms, including those used in MIPD systems, rely on serum creatinine as the primary index of renal function, which inadequately captures the enhanced clearance capacity characteristic of ARC. Once reliable biomarkers for ARC are identified, this information can be integrated into computerized ICU decision-support systems.^66^ However, existing systems retain limited predictive utility for ARC patients because the underlying datasets lack adequate representation of this population.^62^ Until prediction models are developed from cohorts that include substantial numbers of patients with ARC, accurate dosing predictions for this population will remain elusive. Therefore, TDM will remain an essential component for confirming the impact of ARC on antibiotic exposures and guiding dosing adjustments in patients with high clearance rates.^50^

### Conclusions

ARC is an important and often overlooked physiological state that can significantly impact drug dosing, drug efficacy and patient outcomes, especially in the critically ill. Heightened awareness among clinicians, coupled with appropriate diagnostic measures and proactive dose adjustments, is crucial to mitigate the risks associated with drug dosing in ARC. Further research is needed to refine our understanding of this phenomenon, provide better predictive models, easily derived dosing and evidence-based strategies for its optimal management, ultimately aiming to eliminate the danger to dosing for patients with this challenging condition. Finally, current dosing prediction models including MIPD are not derived from enough patients with ARC, and until such time as they use such baseline populations, will not accurately predict dosing requirements in patients with ARC.
