# Correction to: The long walk to a short half-life: the discovery of augmented renal clearance and its impact on antibiotic dosing

**DOI:** 10.1093/jac/dkag205

**Published:** 2026-06-12

This is a correction to: Jeffrey Lipman, Russell E Lewis, The long walk to a short half-life: the discovery of augmented renal clearance and its impact on antibiotic dosing, *Journal of Antimicrobial Chemotherapy*, Volume 80, Issue 12, December 2025, Pages 3367–3374, https://doi.org/10.1093/jac/dkaf378

In the originally published version of this manuscript, there were six instances of a typographical error which saw ‘creatine’ published instead of ‘creatinine’.

In the abstract, a sentence has been corrected to read: “ARC is often undiagnosed unless some form of creatinine clearance is directly measured”.

The caption of Figure 1 has been corrected to read: “Relationship of cefepime and cefpirome clearance versus measured 24 h creatinine clearance in ICU patients. The linear relationship between measured 24 h creatinine clearance (*x*- axis) and calculated drug clearance (*y*-axis) is represented for cefepime (solid line) and cefpirome (dotted line)”.

Under the ‘How prevalent is ARC?’ heading, a sentence has been corrected to read: “While many studies have defined the prevalence of ARC based on timed creatinine clearance, other studies have defined the population of patients with ARC based on low systemic exposures of antibiotics in patients with ‘normal’ serum creatinine”.

Under the ‘Diagnosis of ARC’ heading, a sentence has been corrected to read: “ARC is undiagnosed unless some form of creatinine clearance is directly measured (either via a timed measurement of urine creatinine or less accurately from equations using serum creatinine; see comments below)”.

Additionally, two corrections have been made to Table 1. The reference to the 'Cook et al., 2024.,' study was erroneously omitted and has now been added. In the 'Wang et al., 2023' row, a sentence has been emended to read: “[...] aplastic anaemia more than in those with other haematological diseases due to a higher BMI and a lower GFR” instead of: “[...] plastic anaemia more than in those with other haematological diseases due to a higher BMI and a lower GFR”.

